# Treatment of clinical stage I non-seminoma

**DOI:** 10.1016/j.ajur.2021.03.001

**Published:** 2021-03-06

**Authors:** Christian Winter, Andreas Hiester

**Affiliations:** Department of Urology, University of Duesseldorf, Medical Faculty, Heinrich-Heine-University, Duesseldorf, Germany

**Keywords:** Germ cell tumors, Non-seminomatous germ cell tumors, Active surveillance, Retroperitoneal lymph node dissection

## Abstract

Germ cell cancers are the most common solid tumors among men between 15 and 40 years. Non-seminomatous germ cell tumors (NSGCTs) represent a unique and exclusive cohort of germ cell tumor patients. Non-seminoma can harbor different histologic components. The most commonly found histologies are embryonal cell cancer, teratoma, yolk sack tumor and choriocarcinoma, as well as teratocarcinoma and seminoma, in combination with non-seminomatous germ cell tumors histologic types. The clinical definition of stage I non-seminoma is the absence of metastatic lesions on imaging and normal tumor markers. The cure rate for clinical stage I NSGCT is 99% and this can be achieved by three therapeutic strategies: Active surveillance with treatment at the time of relapse, retroperitoneal lymph node dissection or adjuvant chemotherapy. The balancing of these various strategies should always be based on an individual risk profile of NGSCG patient depending on the lymphovascular invasion of the tumor.

## 1 Introduction

**Figure 1:**
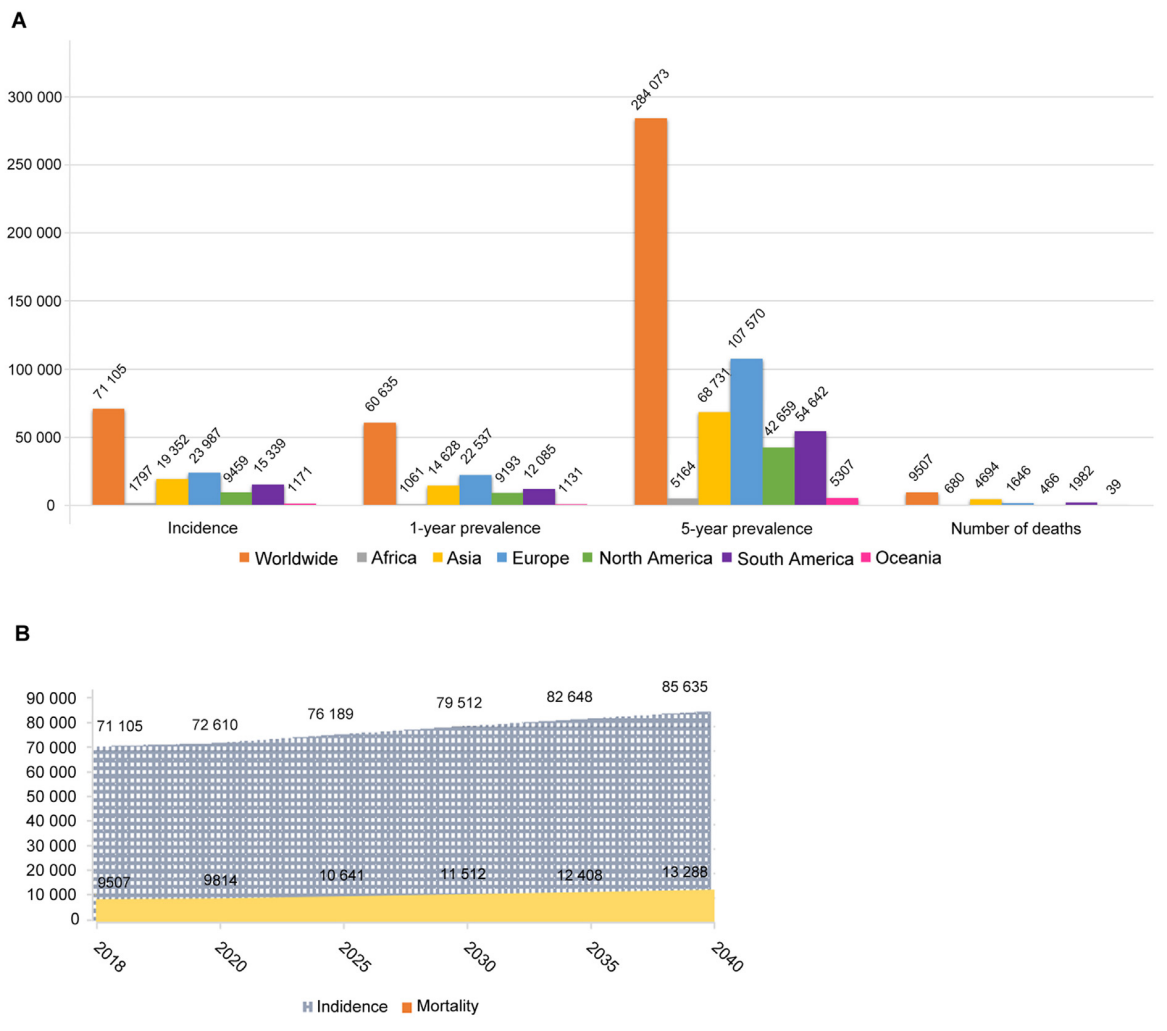
The worldwide incidence for testis cancer Incidence. (A) 1-year and 5-year prevalence and number of deaths in 2018 for testicular cancer; (B) Expected number of incident cases and deaths from 2018 to 2040 for testicular cancer. (GLOBOCAN 2018, https://gco.iarc.fr/).

Germ cell cancers are the most common solid tumors among men between 15 and 40 years. With a worldwide incidence of 70 000 cases, germ cell cancers account just for 1% of all the male tumors [1]. Fig. 1 shows the worldwide incidence for testis cancer in 2018 with a mortality rate of 13.4% (9507 patients). During the last three decades, the incidence of germ cell cancer has increased in industrialized countries of the Northern Hemisphere, although mortality decreased over time [2,3]. This aspect is due to more effective cisplatin-based chemotherapy leading to higher cure rates even in advanced stages [4], whereas the increasing incidence of germ cell cancers cannot be thoroughly explained.

Germ cell cancer is described according to clinical stage, which represents the metastatic spread of the disease. Table 1 shows the stage grouping for non-seminomatous germ cell tumors (NSGCTs) up to stage I. Stage 0, IA IB, and IC represent non-metastatic germ cell cancer; instead stage Is, stage II and stage III represent metastatic germ cell cancer [5,6]. Regardless of any risk factors, stage I testis cancer patients after orchiectomy are often referred to an active surveillance strategy, in order to detect at earliest moment, any possible recurrence. NSGCTs represent a unique and exclusive cohort of germ cell tumor (GCT) patients, as the recurrence rate varies widely from low as 10% up to 50% [[7], [8], [9], [10]]. Irrespectively of the tumor features, it was shown that oncological outcomes were improved in any of the aforementioned groups, when patients were referred to high-volume centers and through integration of multidisciplinary care [[11], [12], [13]].

**Table 1:** Stage grouping for NSGCT (up to Stage I) [5].

Clinical stage	Characteristic			
Stage 0	pTis	N0	M0	S0, SX
Stage I	pT1-pT4	N0	M0	SX
Stage IA	pT1	N0	M0	S0
Stage IB	pT2-pT4	N0	M0	S0
Stage IS	Any pT	N0	M0	S1-3

N0, no lymph node metastasis; M0, no metastasis; S0, no tumor marker elevation; SX, tumor marker elevation; NSGCT, non-seminomatous germ cell tumors.

NSGCTs can harbor different histologic components. The most commonly found histologies are embryonal cell cancer, teratoma, yolk sack tumor and choriocarcinoma, as well as teratocarcinoma and seminoma, in combination with NSGCT histologic types. Rarely teratoma with transformation into somatic-type malignancies such as sarcoma or adenocarcinoma occurs, whereas this special GCT form should be given special attention when making therapy decisions.

**Figure 2:**
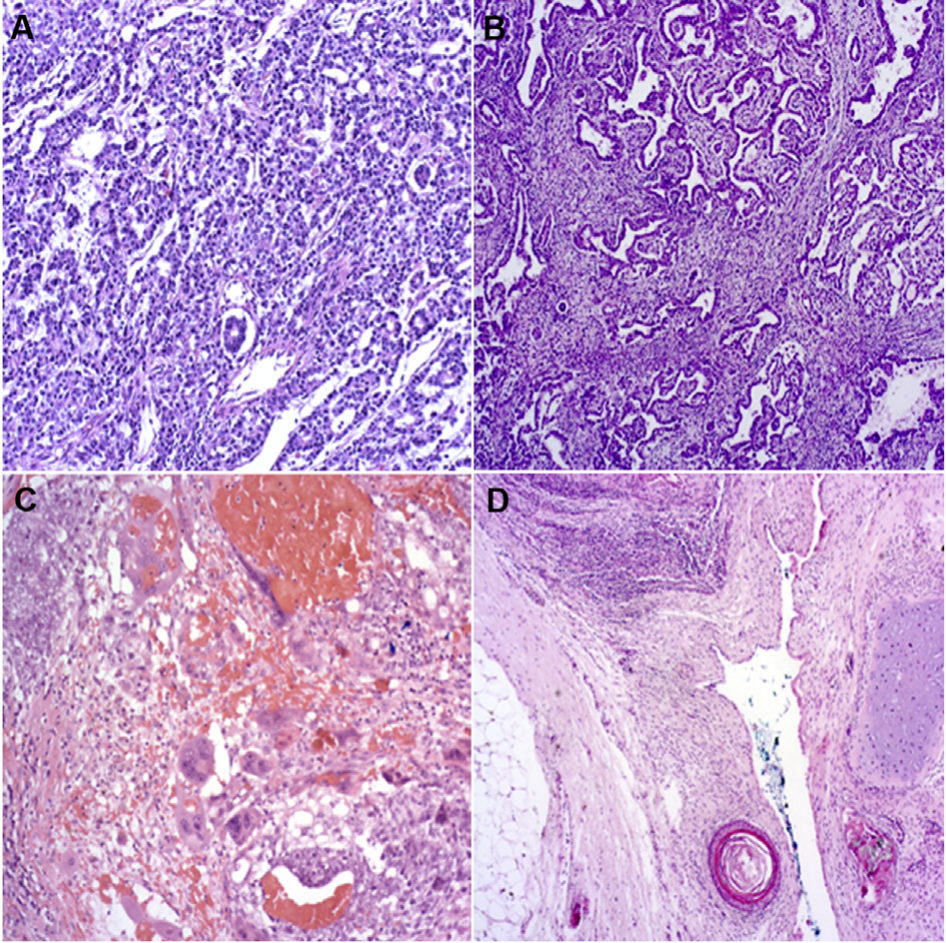
The results of histochemical staining. (A) Microscope slides of yolk sac (10×); (B) embryonal cell carcinoma (5×); (C) choriocarcinoma (10×); (D) teratoma (5×).

Alpha-fetoprotein (AFP) and beta-human chorionic gonadotropin (β-HCG) can be elevated with any entity of tumor. The exact differentiation can only be made by histology report (Fig. 2).

The clinical definition of stage IA and IB is the absence of metastatic lesions on imaging and normalized tumor markers (AFP, HCG, and lactate dehydrogenase [LDH]) after orchiectomy. However, even though in absence of metastasis at imaging, the recurrence rate in stage I NSGCT ranges from 20% to 30%, regardless of risk factors. Non-detectable micrometastases on imaging represent the reason for recurrences in the retroperitoneal area [[7], [8], [9], [10]]. Metastases are most commonly confined to the retroperitoneum, but can also occur simultaneously in retroperitoneal and extraretroperitoneal sites (most commonly lung) or even have skip metastases (most commonly to lung, without retroperitoneal involvement). Skip metastases are more common in non-seminoma than seminoma [14].

If the marker level for AFP or HCG increases or is persistent after orchiectomy and no metastasis is seen in imaging, the patient is in stage CS IS and still has residual disease. To exclude a contralateral GCT, an ultrasound examination of the contralateral testicle must be performed. The treatment of true CS1S NSGCT patients with non-seminoma Stage IS (tumor marker positive) is still controversial, but may be treated with chemotherapy and with follow-up as for CS1B patients [15].

The highest risk of recurrence is registered during the first 2 years of follow-up, although maintaining a 5-year cancer specific survival of 99.7% [8]. Altogether, the median life expectancy of a 30-year-old man diagnosed with stage I NSGCT is 75 years, which is only 2 years less than patients not diagnosed with testicular cancer at all [16]. Even though this excellent survival prognosis is seen across multiple populations in various series, a standardized procedure and recommendation according to risk factors and correct classification may lead to less side effects and an improvement of treatment.

## 2 Risk factors for stage I NSGCTs

Several factors have been evaluated to estimate the risk of recurrence and described in the past [[17], [18], [19]]. The proportion of embryonal carcinoma, the proliferation rate and the lymphovascular invasion [20] have been described as prognostic factors of recurrence [17,19,21]. In multivariate analysis, lymphovascular invasion [20] overruled the other risk factors and is therefore used to stratify stage I NSGCT into “high risk” and “low risk” groups. Patients without lymphovascular invasion (LVI–) on histopathological report of orchiectomy represent the pathological stage pT1 and the clinical stage IA. Patients with lymphovascular invasion (LVI+) on histopathological report of orchiectomy represent the pathological stage pT2 and the clinical stage IB.

High-risk patients without adjuvant therapy carry a risk of recurrence of 27%–50%, while the recurrence rate of low-risk patients is between 12% and 19% [[7], [8], [9], [10]]. Because of this difference, the European Association of Urology (EAU) guidelines for testicular cancer [4], as well as the National Comprehensive Cancer Network (NCCN) guidelines [22] and the German S3 guidelines [23] recommend a risk-adapted treatment of stage I NSGCT.

With promising emerging biomarkers (*e.g.* microRNA), a new approach to risk stratification has emerged in recent months. MicroRNA was first described in the early 1990s and its emergence and importance in cell and cancer function have been growing. Importantly, miRNAs are deregulated in malignancies and can be accurately quantified and measured in the serum through quantitative polymerase chain reaction. MiR-371a-3p was found to have the highest diagnostic sensitivity (88.7%) and specificity of 93.4% for active disease of testicular cancer and correlated with response to treatment [24]. The level of miR-371a-3p correlates with tumor burden and decreases after surgery and/or chemotherapy.

Several studies are currently in process to determine the role of mRNAs, particularly in the CS I NSCGT stage. The goal would be to more accurately predict the presence of microscopic disease in the retroperitoneum over conventional markers and imaging options.

## 3 Treatment options

The cure rate for clinical stage I NSGCT is 99% and this can be achieved by three therapeutic strategies: Active surveillance with treatment at the time of relapse, RPLND with or without adjuvant treatment of pS II disease or a risk-adapted therapy, involving surveillance for low-risk group and adjuvant chemotherapy for high-risk one.

The balancing of the various strategies should always be based on an individual consultation with the patient, which, in accordance with the individual risk profile, includes the advantages and disadvantages of the various therapy strategies and takes into account the individual life situation of the patient. Before initiation of adjuvant therapy either chemotherapy or surgery, advice on fertility-protective measures is required and semen cryoconservation should be offered [23].

If only teratoma is histologically detected in the primary tumor in GCT patient, important management implications are required. Teratoma typically grows slowly or may be indolent. However, teratoma contains many genetic abnormalities and therefore underlies genetic instability. This may lead to uncontrollable growth (“growing teratoma syndrome”) or transformation into somatic-type malignancies such as sarcoma or adenocarcinoma. In several study, chemotherapy showed no efficacy for teratoma with somatic-type malignant transformation [[25], [26], [27]]. Teratoma is universally resistant to chemotherapy and only curable by surgical resection, so RPLND is recommended for these patients to remove any retroperitoneal metastases that may exist and reduce the risk of relapse.

## 4 Risk-adapted treatment

Risk-adapted treatment is based on the discriminative power of the risk factor LVI. As patients with LVI+ (“high risk”) will have a recurrence in up to 50%, patients with LVI– (“low risk”) will only suffer from recurrence in 14%. Due to this difference, risk-adapted treatment can be recommended, with the offer for adjuvant treatment in high-risk patients and active surveillance in low-risk patients.

### 4.1 Chemotherapy

The most common and recommended regime for patients with stage I “high-risk” NSCCT is the administration of one cycle of chemotherapy with Bleomycin, Etoposide and Cisplatin (BEP). The recommended schedule by the EAU guidelines for BEP is:

Cisplatin 20 mg/m^2^*i.v.* (30 min) Days 1–5

Etoposide 100 mg/m^2^*i.v.* (60 min) Days 1–5

Bleomycin 30 mg *i.v.* (bolus) Days 2, 8, 15

Platinum complexes are clinically used to induce cell death. Cisplatin (CDDP) is one of the most potent drugs in the treatment of germ cell cancer [28]. Depending on its concentration, platinum is cytotoxic by interfering with DNA-repair mechanisms, and induces apoptosis mediated by various signaling pathways like calcium signaling, death receptor signaling and mitochondrial signaling [29]. Unfortunately, the use of cisplatin is limited due to its side effects and its cytotoxic effects on healthy tissue. Patients should be always informed about side effects and drug toxicities. Key management principles and strategies to avoid side effects include renal protection by hydration, antiemetics and strict monitoring of the blood cells. A myelosuppression leads to a decrease in red and white blood cell count and decreasing platelets.

Etoposide was developed in the late 1970s. Hande [30], Loike and Horwitz [31] demonstrated anti-tumor activity for etoposide leads to single-strand and double-strand DNA breaks in cells incubated with etoposide by inhibiting topoisomerase II.

The third key drug used for chemotherapy in the BEP-scheme is bleomycin. Bleomycin is less myelotoxic than cisplatin and etoposide but a severe side effect that is associated with bleomycin is lung fibrosis [32]. This pulmonary toxicity seems to be caused by an inflammatory reaction of epithelial cells of the lung. The mechanisms involved are not fully understood yet but seem to include transforming growth factor beta 1, tumor necrosis factor, interleukins and chemokines [33].

In Table 2 and the following section, the most common side effects are named and listed.

**Table 2:** Dose-related and non-dose related toxicities of BEP chemotherapy [58].

Dose-related	Non-dose related	Unknown
Infertility	Febrile neutropenia	Second malignancy
Peripheral neuropathy	Alopecia	Cardiovascular disease
Ototoxicity	Nausea/vomiting	–
Raynaud's phenomena	–	–
Fatigue	–	–
Skin toxicity	–	–
Avascular necrosis hip	–	–
Pneumonitis/Lung fibrosis	–	–
Renal damage	–	–
Anaemia	–	–
Metabolic syndrome	–	–

–, no available; BEP, bleomycin, etoposide and cisplatin.

### 4.2 Cisplatin

- Cisplatin has an high emetogenic potential, which can usually efficiently be treated with 5-HT3-Antagonists (*e.g.* granisetron) prior to administration of cisplatin and NK-1-Antagonist combined with dexamethasone on Day 1–3 of the chemotherapy.
- Another well-known side effect is renal function impairment. This side effect can be prevented by forced diuresis and minimum fluid volume administration (at least 2.5 L per day) [34].
- In general, cisplatin has a great potential of hearing loss, especially in young patients [35]. In 2006, Rademaker-Lakhai [36] could show that ototoxicity is associated with dose-escalation. Hearing-loss and tinnitus vary individually and normally not occur after one cycle of BEP.
- Myelosuppression also is a rare and mostly not severe phenomenon after one cycle of BEP. It reaches its Nadir at Day 10–14.
- Cisplatin is cardiotoxic. Antineoplastic treatment might result in alterations of the electrocardiogram. Especially a prolongation of the QT-interval has been described [37]. Therefore a cardiological assessment is required in order to reduce the risk of exposure vulnerable individuals to cisplatin, such as those with congenital long-QT-syndrome [38].
- Anaphylactic reactions are rare.

### 4.3 Etoposide

- Nausea and vomiting have been seen in 25% of patients.
- Myelosuppression is the major side effect.
- Alopecia.

### 4.4 Bleomycin

- Pneumonitis and lung fibrosis are the most important side effects of Bleomycin. The risk of these complications increases with the total dose employed. In cases of acute dyspnea, if infectious complications of the lung, except bronchitis, occur, Bleomycin should be stopped. In case of surgical procedures or respiratory insufficiency, oxygen therapy should be offered with caution because of the increased risk of oxygen toxicity to the lung in Bleomycin treated patients.
- Fever, rigors, and skin toxicity may occur as well.

In 1985, the Austrian group started to administer BEP as adjuvant treatment in 42 patients. Long-term outcomes 11 years later resulted with two relapses and one patient who had died of disease [39]. In the same year, Cullen et al. [40] published their experience with two cycles BEP in 114 high-risk patients, with a follow-up of more than 2 years and a recurrence rate of only 1.8%. Since 50% of high-risk patients are still overtreated by this approach, long-term toxicity assessment is crucial. Reports on late toxicities of cisplatin-based chemotherapy show an increased rate of cardiovascular disease (1.4–7.1 fold) and secondary malignancies (1.8–2.1 fold) [41]. Renal toxicity, impaired hearing and Raynaud's disease are described as well [41]. In order to avoid toxicity, administration of one cycle of BEP is the preferred treatment instead of using two cycles of BEP.

First, Oliver et al. [42] presented data of the UK National Database of testicular cancer patients, where high-risk patients received one or two cycles of BEP, while low-risk patients were treated with active surveillance. The results showed that patient treated with one and two cycles of chemotherapy had lower recurrence rates compared to active surveillance patients [42]. In a prospective, non-randomized, risk-adapted trial of the Swedish and Norwegian Testicular Cancer Group (SWENOTECA), high-risk patients were treated with one cycle of BEP, while low-risk patients could choose between one cycle of BEP or active surveillance. In all patients treated with BEP (independent of risk group) the recurrence rate was 2.3%, where the recurrence rate for high-risk patients was 3.2% and for low-risk patients 1.6%.

Overall, the reduction of adjuvant chemotherapy by risk-adapted treatment to one cycle of BEP appears to improve the benefit–risk profile for patients: Lower toxicity while still maintaining low recurrence risk.

The draw-back of chemotherapy in terms of long-term toxicities and still a high rate of overtreated patients has to be taken into account. With three and more cycles of BEP, relevant toxicities and a significantly higher rate of secondary malignancies have been published. Up to date, no conclusive data on the long-term toxicity of one course of BEP are available. Only in a Swiss study with few patients, it was shown measurable rates of tinnitus and peripheral neuropathy 10 years after one course of chemotherapy with BEP [43].

Evaluations of other possible chemotherapy regime (*e.g.* CVB—cisplatin, vinblastine and bleomycin or BOP—bleomycin, vincristine and cisplatin) in treatment of NSGCT CS I patients showed no significant differences in oncological outcome but unacceptably high toxicities [44,45]. If there is a real contraindication to BEP chemotherapy, then the therapy option of RPLND or active surveillance should be chosen.

## 5 Role of RPLND

Historically, RPLND has been the classical treatment option for this disease stage. Advantages included correct pathological staging, low short-term morbidity and exclusively pulmonary recurrence rates of 8% [46] (Table 3). Since reduction of long-term toxicity and overtreatment should be aimed for testicular cancer patients, Albers et al. [46] investigated the role of RPLND versus BEP. In this German phase-III study, 380 patients were randomized to receive one cycle of BEP or a nerve-sparing RPLND. The recurrence rate was 7.8% for RPLND and 1% for BEP. However, it should be noted that no risk stratification was performed in this study [46].

**Table 3:** Intraoperative, early and late complications of retroperitoneal lymph node dissection [47].

Complication	Percent (%)
Retrograde ejaculation	6.7
Wound infection	5.4
Ileus	2.1
Chylous ascites	2.1
Vascular lesion with intraoperative repair	2.0
Lymphocele	1.7
Hydronephrosis	1.2
Ventral hernia	0.8
Stomach ulcer	0.8
Lung embolism	0.8
Keloid	0.8
Bleeding	0.8
Urinary tract infection	0.4
Ureteral lesion with ureteral stent insertion	0.4
Small bowel obstruction	0.4
Renal artery lesion with Nephrectomy	0.4
Pneumonia	0.4
Large bowel lesion with intraoperative repair	0.4
Inferior mesenteric artery lesion with hemicolectomy and temporary colostomy	0.4
Epididymitis	0.4
Deep vein thrombosis	0.4

Further studies showed that RPLND alone could prevent recurrence and minimize late relapses, where most patients could avoid the immediate and late toxicity of a chemotherapy [[47], [48], [49]].

Since RPLND showed a higher than expected recurrence rate than one cycle of BEP in Albers Phase III study, the recommended standard of care for high-risk patients seems to be chemotherapy. Nevertheless, RPLND hold its place in the adjuvant treatment of stage I NSGCT. Especially RPLND is the recommended treatment in patients with teratomas and somatic transformation of the primary tumor [47,50]. Due to nerve-sparing approach and preservation of antegrade ejaculation, this procedure should be performed only at a specialized center [51,52] (Table 4, Table 5).

**Table 4:** Follow-up clinical stage I NSGCT without risk factors according to the NCCN guidelines [22].

NCCN guidelines	Year 1	Year 2	Year 3	Year 4	Year 5
Physical examination	Every 2 months	Every 3 months	Every 4–6 months	Every 6 months	Annually
Tumor marker	Every 2 months	Every 3 months	Every 4–6 months	Every 6 months	annually
Chest X-ray	2 times (at month 4 and 12)	Annually	Annually	Annually	Annually
Abdominopelvic CT scan	2–3 times	Annually	Annually	No scan	No scan

NSGCT, non-seminomatous germ cell tumor; NCCN, National Comprehensive Cancer Network; CT, computed tomography.

**Table 5:** Follow-up clinical stage I NSGCT with risk factors according to the NCCN guidelines [22].

NCCN guideline	Year 1	Year 2	Year 3	Year 4	Year 5
Physical examination	Every 2 months	Every 3 months	Every 4–6 months	Every 6 months	Annually
Tumor marker	Every 2 months	Every 3 months	Every 4–6 months	Every 6 months	Annually
Chest X-ray	Every 4 months	Every 4–6 months	Every 6 months	Annually	Annually
Abdominopelvic CT scan	Every 4 months	Every 4–6 months	Every 6 months	Annually	Annually

NSGCT, non-seminomatous germ cell tumor; NCCN, National Comprehensive Cancer Network; CT, computed tomography.

The role of minimally invasive RPLND such as a laparoscopic or robotic-assisted RPLND in the management of GCT is still controversial, but in the last years increasingly advocating. Multiple cohorts have demonstrated feasibility and safety of minimally invasive RPLND [20,50,53,54]. While laparoscopic and robotic-assisted RPLND have a promising future in the management for primary low-stage NSGCT, more prospective studies are needed before supplanting the open RPLND as the gold standard approach.

The role of chemotherapy after RPLND in GCT patients is controversial. The option for adjuvant treatment depends on pathologic findings in the resected specimen. For patients with no viable cancer at resected specimen or low-volume nodal metastases (pN1) with negative tumor markers and a complete resection, RPLND offers a greater than 90% cure rates [55]. However, in patients with high-volume lymph nodes metastasis (pN2), an adjuvant chemotherapy should be discussed. Several randomized trials showed a significant reduction in relapse but no difference in overall survival [56,57]. It should be noted the overall survival rates were similar due to the effectiveness of salvage chemotherapy, when it is needed in case of recurrence.

## 6 Active surveillance

**Figure 3:**
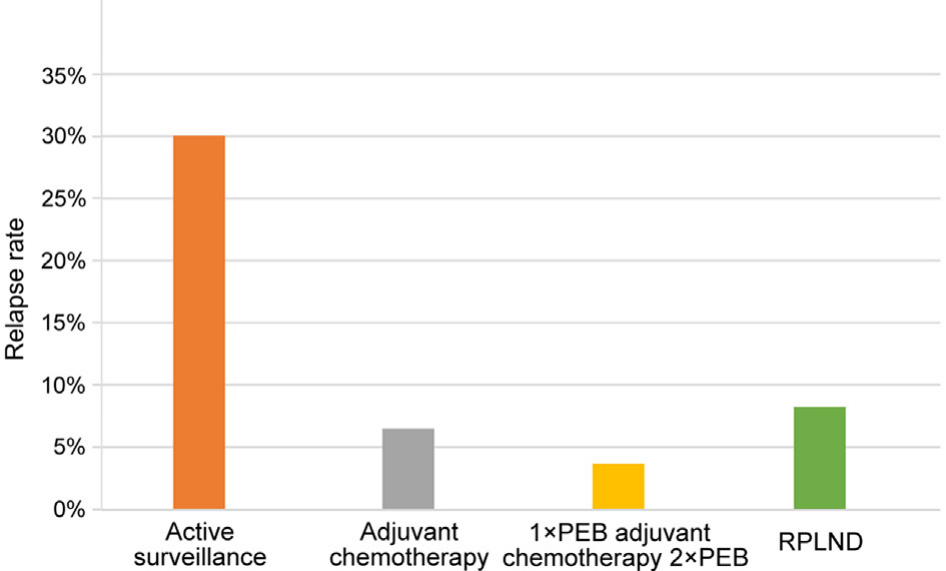
Relapse rates according to different treatment strategies for NSGCT stage I [39,40]. RPLND, retroperitoneal lymph node dissection; NSGCT, non-seminomatous germ cell tumors; BEP, bleomycin, etoposide, and cisplatin.

Active surveillance plays a crucial role in risk-adapted treatment of stage I NSGCT patients. Since 50% of the high-risk population and up to 88% of the low-risk population are overtreated with chemotherapy or RPLND, a defined surveillance protocol is crucial for monitoring the patients with stage I NSGCT. Regardless of risk stratification, active surveillance in stage I NSGCT carries a 30% risk of recurrence (Fig. 3). It is known that 80% of relapses occur during the first year, 12% during the second, 6% during third year and only 1% in Year 4 and 5 after primary treatment with orchiectomy. Survival figures are not compromised if active surveillance is performed and treatment tailored to patients with relapse only. In the last years, the number of CT scans has been dramatically reduced. The current EAU guidelines recommend actually two CT scans at 3- and 12-month during follow-up [4], while the NCCN guidelines recommend abdominal and pelvic CT scan every 4–6 months for the first, twice for the second and annually for the third year in patients with low-risk stage I NSGCT and for patients with high-risk stage I NSGCT every 4 months for the first, every 4–6 month for the second, every 6 month for the third and for the fifth and sixth year annually [22] (Table 5).

Finally, patients have to decide which treatment option fits best to their personal circumstances. A thorough informed consent should be reached after explaining pros and cons of every treatment option possible. Survival rates are not different and overall very high with each approach, therefore, individual issues like time off work, contraindications or problems with chemotherapy, acceptance of long-term toxicities, and psychological burden play a major role in the decision-making process.

## 7 Conclusion

The recurrence rate in stage I NSGCT patients varies between 12% and 50%. Due to this wide range of recurrence rate, patients have to be accurately and conscientiously informed. Throughout the different international guidelines, risk factors are taken into account for decision making.

EAU guidelines [4], AUA guidelines [14], German S3 guidelines [23] and NCCN guidelines [22] all recommend surveillance in case of NSGCT stage I without risk factors (pT1, LVI–) as preferred treatment option if the patient is compliant and the circumstances allow it. Whether circumstances allow active surveillance or not, it is suggested to offer one cycle of BEP. RPLND should be reserved for patient who cannot undergo active surveillance or chemotherapy.

**Figure 4:**
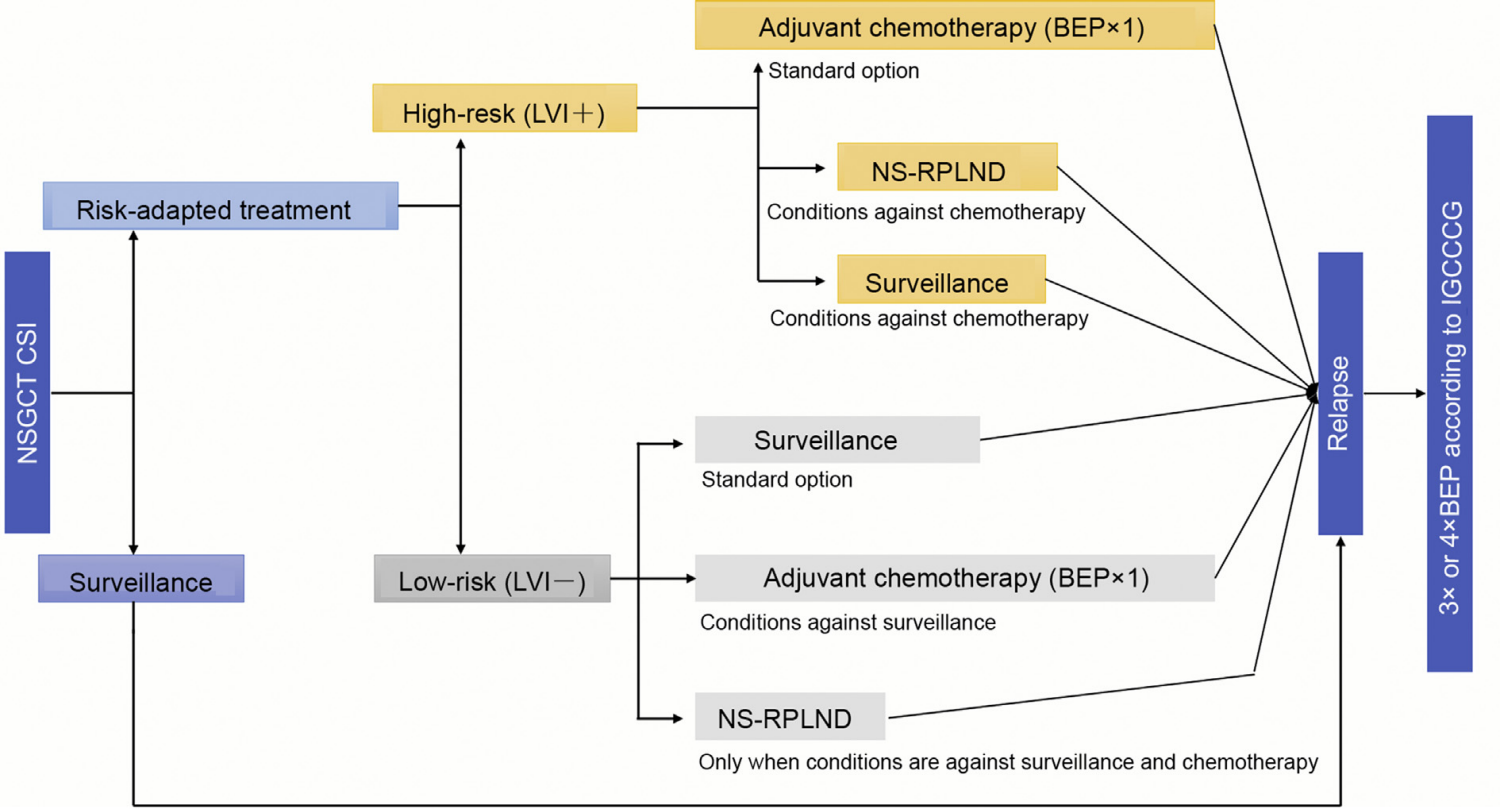
Flowsheet of treatment of NSGCT stage I (modified after [4]). NSGCT, non-seminomatous germ cell tumors; LVI+, lymphovascular invasion; LVI−, no lymphovascular invasion; IGCCCG, International Germ Cell Cancer Collaborative Group; BEP, bleomycin, etoposide, and cisplatin; NS-RPLND, nerve-sparing retroperitoneal lymph node dissection; NSGCT, non-seminomatous germ cell tumor; CSI, clinical stage I.

In case of presence of risk factors (pT2, LVI+), the recommendations of AUA, NCCN and EAU differ slightly. The AUA guidelines state that clinicians should recommend surveillance, RPLND, or one or two cycles of BEP based on shared decision-making [14]. The NCCN guidelines recommend surveillance or one cycle of BEP or nerve-sparing RPLND [22]. The EAU guidelines and the German S3 guidelines recommend to inform patients with stage I NSGCT about all possible adjuvant treatment options after orchiectomy, including active surveillance, adjuvant chemotherapy and RPLND, with regard to recurrence rates, side effects, long term toxicity and risk-adapted treatment, based on vascular invasion (LVI+/−). In high-risk patients (pT2, LVI+), the EAU guidelines and German S3 guideline recommend one course of BEP. In patients who are not willing to undergo chemotherapy, active surveillance should be performed. RPLND should only be offered in special cases, since it is less effective than chemotherapy. Fig. 4 shows a detailed flow-chart of treatment of NSGCT modified after the EAU Guidelines.

In case of recurrence after adjuvant treatment or active surveillance, the patient should undergo three or four cycles of BEP according to the International Germ Cell Cancer Collaborative Group classification [4].

## Authors contribution

*Study design:* Christian Winter.

*Data acquisition:* Christian Winter, Andreas Hiester.

*Data analysis:* Andreas Hiester.

*Drafting of manuscript:* Christian Winter, Andreas Hiester.

*Critical revision of the manuscript:* Christian Winter.

## Conflicts of interest

The authors declare no conflict of interest.

## References

[1] Shanmugalingam T., Soultati A., Chowdhury S., Rudman S., Van Hemelrijck M. (2013). Global incidence and outcome of testicular cancer. Clin Epidemiol.

[2] Le Cornet C., Lortet-Tieulent J., Forman D., Béranger R., Flechon A., Fervers B. (2014). Testicular cancer incidence to rise by 25% by 2025 in Europe? Model-based predictions in 40 countries using population-based registry data. Eur J Canc.

[3] Ghazarian A.A., Trabert B., Devesa S.S., McGlynn K.A. (2015). Recent trends in the incidence of testicular germ cell tumors in the United States. Andrology.

[4] Albers P., Albrecht W., Algaba F., Bokemeyer C., Cohn-Cedermark G., Fizazi K. (2015). Guidelines on testicular cancer: 2015 update. Eur Urol.

[5] Wittekind C. (2014). [TNM classification of testicular tumors. Definitions and prerequisites for correct application]. Pathologe.

[6] Winter C., Albers P. (2011). Testicular germ cell tumors: pathogenesis, diagnosis and treatment. Nat Rev Endocrinol.

[7] Kollmannsberger C., Moore C., Chi K.N., Murray N., Daneshmand S., Gleave M. (2010). Non-risk-adapted surveillance for patients with stage I nonseminomatous testicular germ-cell tumors: diminishing treatment-related morbidity while maintaining efficacy. Ann Oncol.

[8] Kollmannsberger C., Tandstad T., Bedard P.L., Cohn-Cedermark G., Chung P.W., Jewett M.A. (2015). Patterns of relapse in patients with clinical stage I testicular cancer managed with active surveillance. J Clin Oncol.

[9] Sturgeon J.F., Moore M.J., Kakiashvili D.M., Duran I., Anson-Cartwright L.C., Berthold D.R. (2011). Non-risk-adapted surveillance in clinical stage I nonseminomatous germ cell tumors: the Princess Margaret Hospital's experience. Eur Urol.

[10] Daugaard G., Gundgaard M.G., Mortensen M.S., Agerbæk M., Holm N.V., Rørth M. (2014). Surveillance for stage I nonseminoma testicular cancer: outcomes and long-term follow-up in a population-based cohort. J Clin Oncol.

[11] Collette L., Sylvester R.J., Stenning S.P., Fossa S.D., Mead G.M., de Wit R. (1999). Impact of the treating institution on survival of patients with “poor-prognosis” metastatic nonseminoma. European Organization for Research and Treatment of Cancer Genito–Urinary Tract Cancer Collaborative Group and the Medical Research Council Testicular Cancer Working Party. J Natl Cancer Inst.

[12] Nayan M., Jewett M.A., Anson-Cartwright L., Bedard P.L., Moore M., Chung P. (2016). The association between institution at orchiectomy and outcomes on active surveillance for clinical stage I germ cell tumours. Can Urol Assoc J.

[13] Adra N., Althouse S.K., Liu H., Brames M.J., Hanna N.H., Einhorn L.H. (2016). Prognostic factors in patients with poor-risk germ-cell tumors: a retrospective analysis of the Indiana University experience from 1990 to 2014. Ann Oncol.

[14] Stephenson A., Eggener S.E., Bass E.B., Chelnick D.M., Daneshmand S., Feldman D. (2019). Diagnosis and treatment of early stage testicular cancer: AUA guideline. J Urol.

[15] Davis B.E., Herr H.W., Fair W.R., Bosl G.J. (1994). The management of patients with nonseminomatous germ cell tumors of the testis with serologic disease only after orchiectomy. J Urol.

[16] Capocaccia R., Gatta G., Dal Maso L. (2015). Life expectancy of colon, breast, and testicular cancer patients: an analysis of US-SEER population-based data. Ann Oncol.

[17] Albers P., Ulbright T.M., Albers J., Miller G.A., Orazi A., Crabtree W.N. (1996). Tumor proliferative activity is predictive of pathological stage in clinical stage A nonseminomatous testicular germ cell tumors. J Urol.

[18] Albers P., Siener R., Kliesch S., Weissbach L., Krege S., Sparwasser C. (2003). Risk factors for relapse in clinical stage I nonseminomatous testicular germ cell tumors: results of the German Testicular Cancer Study Group Trial. J Clin Oncol.

[19] Heidenreich A., Schenkmann N.S., Sesterhenn I.A., Mostofi F.K., McCarthy W.F., Heidenreich B. (1997). Immunohistochemical expression of Ki-67 to predict lymph node involvement in clinical stage I nonseminomatous germ cell tumors. J Urol.

[20] Nicolai N., Tarabelloni N., Gasperoni F., Catanzaro M., Stagni S., Torelli T. (2018). Laparoscopic retroperitoneal lymph node dissection for clinical stage I nonseminomatous germ cell tumors of the testis: safety and efficacy analyses at a high volume center. J Urol.

[21] Ondrus D., Matoska J., Belan V., Kausitz J., Goncalves F., Hornak M. (1998). Prognostic factors in clinical stage I nonseminomatous germ cell testicular tumors: rationale for different risk-adapted treatment. Eur Urol.

[22] Motzer R.J., Jonasch E., Agarwal N., Beard C., Bhayani S., Bolger G.B. (2015). Testicular cancer, version 2.2015. J Natl Compr Canc Netw.

[23] Leitlinienprogramm Onkologie (Deutsche Krebsgesellschaft, Deutsche Krebshilfe, AWMF): S3-Leitlinie Diagnostik, Therapie und Nachsorge der Keimzelltumoren des Hodens, Langversion 0.1 (Konsultationsfassung), 2018 AWMF Registernummer: 043/049OL. https://www.leitlinienprogramm-onkologie.de/leitlinien/hodentumoren.

[24] Dieckmann K.P., Radtke A., Geczi L., Matthies C., Anheuser P., Eckardt U. (2019). Serum levels of microRNA-371a-3p (M371 test) as a new biomarker of testicular germ cell tumors: results of a prospective multicentric study. J Clin Oncol.

[25] Donadio A.C., Motzer R.J., Bajorin D.F., Kantoff P.W., Sheinfeld J., Houldsworth J. (2003). Chemotherapy for teratoma with malignant transformation. J Clin Oncol.

[26] El Mesbahi O., Terrier-Lacombe M.J., Rebischung C., Theodore C., Vanel D., Fizazi K. (2007). Chemotherapy in patients with teratoma with malignant transformation. Eur Urol.

[27] Logothetis C.J., Samuels M.L., Trindade A., Johnson D.E. (1982). The growing teratoma syndrome. Cancer.

[28] Desoize B., Madoulet C. (2002). Particular aspects of platinum compounds used at present in cancer treatment. Crit Rev Oncol Hematol.

[29] Ghosh S. (2019). Cisplatin: the first metal based anticancer drug. Bioorg Chem.

[30] Hande K.R. (1998). Etoposide: four decades of development of a topoisomerase II inhibitor. Eur J Canc.

[31] Loike J.D., Horwitz S.B. (1976). Effect of VP-16-213 on the intracellular degradation of DNA in HeLa cells. Biochemistry.

[32] Kawai K., Akaza H. (2003). Bleomycin-induced pulmonary toxicity in chemotherapy for testicular cancer. Expert Opin Drug.

[33] Della Latta V., Cecchettini A., Del Ry S., Morales M.A. (2015). Bleomycin in the setting of lung fibrosis induction: from biological mechanisms to counteractions. Pharmacol Res.

[34] Momekov G., Ferdinandov D., Bakalova A., Zaharieva M., Konstantinov S., Karaivanova M. (2006). *In vitro* toxicological evaluation of a dinuclear platinum(II) complex with acetate ligands. Arch Toxicol.

[35] Knoll C., Smith R.J., Shores C., Blatt J. (2006). Hearing genes and cisplatin deafness: a pilot study. Laryngoscope.

[36] Rademaker-Lakhai J.M., Crul M., Zuur L., Baas P., Beijnen J.H., Simis Y.J. (2006). Relationship between cisplatin administration and the development of ototoxicity. J Clin Oncol.

[37] Keller G.A., Ponte M.L., Di Girolamo G. (2010). Other drugs acting on nervous system associated with QT-interval prolongation. Curr Drug Saf.

[38] Florea A.M., Busselberg D. (2011). Cisplatin as an anti-tumor drug: cellular mechanisms of activity, drug resistance and induced side effects. Cancers (Basel).

[39] Pont J., Albrecht W., Postner G., Sellner F., Angel K., Holtl W. (1996). Adjuvant chemotherapy for high-risk clinical stage I nonseminomatous testicular germ cell cancer: long-term results of a prospective trial. J Clin Oncol.

[40] Cullen M.H., Stenning S.P., Parkinson M.C., Fossa S.D., Kaye S.B., Horwich A.H. (1996). Short-course adjuvant chemotherapy in high-risk stage I nonseminomatous germ cell tumors of the testis: a Medical Research Council report. J Clin Oncol.

[41] van den Belt-Dusebout A.W., de Wit R., Gietema J.A., Horenblas S., Louwman M.W., Ribot J.G. (2007). Treatment-specific risks of second malignancies and cardiovascular disease in 5-year survivors of testicular cancer. J Clin Oncol.

[42] Oliver R.T., Ong J., Shamash J., Ravi R., Nagund V., Harper P. (2004). Long-term follow-up of Anglian Germ Cell Cancer Group surveillance versus patients with Stage 1 nonseminoma treated with adjuvant chemotherapy. Urology.

[43] Vidal A.D., Thalmann G.N., Karamitopoulou-Diamantis E., Fey M.F., Studer U.E. (2015). Long-term outcome of patients with clinical stage I high-risk nonseminomatous germ-cell tumors 15 years after one adjuvant cycle of bleomycin, etoposide, and cisplatin chemotherapy. Ann Oncol.

[44] Tandstad T., Cohn-Cedermark G., Dahl O., Stierner U., Cavallin-Stahl E., Bremnes R.M. (2010). Long-term follow-up after risk-adapted treatment in clinical stage 1 (CS1) nonseminomatous germ-cell testicular cancer (NSGCT) implementing adjuvant CVB chemotherapy. A SWENOTECA study. Ann Oncol.

[45] Dearnaley D.P., Fossa S.D., Kaye S.B., Cullen M.H., Harland S.J., Sokal M.P.J. (2005). Adjuvant bleomycin, vincristine and cisplatin (BOP) for high-risk stage I non-seminomatous germ cell tumours: a prospective trial (MRC TE17). Br J Canc.

[46] Albers P., Siener R., Krege S., Schmelz H.U., Dieckmann K.P., Heidenreich A. (2008). Randomized phase III trial comparing retroperitoneal lymph node dissection with one course of bleomycin and etoposide plus cisplatin chemotherapy in the adjuvant treatment of clinical stage I Nonseminomatous testicular germ cell tumors: AUO trial AH 01/94 by the German Testicular Cancer Study Group. J Clin Oncol.

[47] Heidenreich A., Albers P., Hartmann M., Kliesch S., Kohrmann K.U., Krege S. (2003). Complications of primary nerve sparing retroperitoneal lymph node dissection for clinical stage I nonseminomatous germ cell tumors of the testis: experience of the German Testicular Cancer Study Group. J Urol.

[48] Foster R.S. (2004). Modified retroperitoneal lymphadenectomy. BJU Int.

[49] Al-Ahmadie H.A., Carver B.S., Cronin A.M., Olgac S., Tickoo S.K., Fine S.W. (2013). Primary retroperitoneal lymph node dissection in low-stage testicular germ cell tumors: a detailed pathologic study with clinical outcome analysis with special emphasis on patients who did not receive adjuvant therapy. Urology.

[50] Neyer M., Peschel R., Akkad T., Springer-Stöhr B., Berger A., Bartsch G. (2007). Long-term results of laparoscopic retroperitoneal lymph-node dissection for clinical stage I nonseminomatous germ-cell testicular cancer. J Endourol.

[51] Hiester A., Nini A., Fingerhut A., Siemer R.G., Winter C., Albers P. (2018). Preservation of ejaculatory function after postchemotherapy retroperitoneal lymph node dissection (PC-RPLND) in patients with testicular cancer: template vs. Bilateral resection. Front Surg.

[52] Lusch A., Albers P. (2017). Residual tumor resection (RTR). World J Urol.

[53] Schwen Z.R., Gupta M., Pierorazio P.M. (2018). A review of outcomes and technique for the robotic-assisted laparoscopic retroperitoneal lymph node dissection for testicular cancer. Adv Urol.

[54] Nelson J.B., Chen R.N., Bishoff J.T., Oh W.K., Kantoff P.W., Donehower R.C. (1999). Laparoscopic retroperitoneal lymph node dissection for clinical stage I nonseminomatous germ cell testicular tumors. Urology.

[55] Williams S.D., Stablein D.M., Einhorn L.H., Muggia F.M., Weiss R.B., Donohue J.P. (1987). Immediate adjuvant chemotherapy versus observation with treatment at relapse in pathological stage II testicular cancer. N Engl J Med.

[56] Motzer R.J., Sheinfeld J., Mazumdar M., Bajorin D.F., Bosl G.J., Herr H. (1995). Etoposide and cisplatin adjuvant therapy for patients with pathologic stage II germ cell tumors. J Clin Oncol.

[57] Weissbach L., Bussar-Maatz R., Flechtner H., Pichlmeier U., Hartmann M., Keller L. (2000). RPLND or primary chemotherapy in clinical stage IIA/B nonseminomatous germ cell tumors? Results of a prospective multicenter trial including quality of life assessment. Eur Urol.

[58] Fung C., Vaughn D.J. (2011). Complications associated with chemotherapy in testicular cancer management. Nat Rev Urol.

